# Items

**Published:** 1907-02

**Authors:** 


### ITEMS.

The Antikamnia calendar for 1907 is an exquisite bit of art work entitled "The Convalescent." The picture is a reproduction of the painting by Miss Magnus which was exhibited in the Manchester Academy.

The State Civil Service Commission will hold examinations in all parts of the State February 23, 1907. Among the positions to be filled are, apothecary, $540 to $900 and maintenance; homeopathic pharmacist, $900 and maintenance; pharmacist, Erie County service, $720; physician, sixth grade, $900 and maintenance; pupil nurse, Erie County Hospital, $10 a month and maintenance.

The last day for filing applications for these positions is February 18th. Full information and application forms for any of these examinations may be obtained by addressing Charles S. Fowler, Chief Examiner of the Commission at Albany.

New Regulations Proposed for the Health Department of Buffalo.—The charter revision committee, through its subcommittee, has drafted resolutions governing the health department. The regulations provide that the health commissioner is to be appointed by the mayor (as he is now), and must be a reputable, licensed physician with actual experience in practice of not less than seven years. The commissioner is to appoint his assistants and, with the council's concurrence, shall fix their salaries. In time of "great and imminent peril to the public health," the commissioner of health may take such measures for safety as he deems necessary. As at present he will have supervision over dead bodies, and the registration of births, marriages and deaths. It is also provided that he shall make rules and regulations for enforcement of all laws and ordinances for the protection of public health and care of vital statistics. One section not only authorizes but requires him to prepare ordinances for licensing all persons offering to give massage and medicinal baths, and all persons and organizations advertising to practise medicine in any of its branches. He is further empowered to enforce all health ordinances and is given greater power over tenements than is provided for in the present charter. All drainage, plumbing, etc., in all kinds of buildings shall be subject to the commissioner's approval and if not prohibited by statute or law, the commissioner is empowered to permit or prohibit the building of dwellings to be used by more than two families, of livery stables, barns in which live stock is to be kept, slaughterhouses or rendering plants: or the transforming of present buildings into structures for any of such uses.

